# Mosaic Hemagglutinin-Based Whole Inactivated Virus Vaccines Induce Broad Protection Against Influenza B Virus Challenge in Mice

**DOI:** 10.3389/fimmu.2021.746447

**Published:** 2021-09-16

**Authors:** Yonghong Liu, Shirin Strohmeier, Irene González-Domínguez, Jessica Tan, Viviana Simon, Florian Krammer, Adolfo García-Sastre, Peter Palese, Weina Sun

**Affiliations:** ^1^Department of Microbiology, Icahn School of Medicine at Mount Sinai, New York, NY, United States; ^2^Department of Medicine, Icahn School of Medicine at Mount Sinai, New York, NY, United States; ^3^Global Health Emerging Pathogens Institute, Icahn School of Medicine at Mount Sinai, New York, NY, United States; ^4^Department of Pathology, Molecular and Cell-Based Medicine, Icahn School of Medicine at Mount Sinai, New York, NY, United States; ^5^The Tisch Cancer Institute, Icahn School of Medicine at Mount Sinai, New York, NY, United States

**Keywords:** influenza B virus, whole inactivated virus vaccine, immuno-subdominant epitopes, broad protection, universal influenza B vaccine

## Abstract

Influenza viruses undergo antigenic changes in the immuno-dominant hemagglutinin (HA) head domain, necessitating annual re-formulation of and re-vaccination with seasonal influenza virus vaccines for continuing protection. We previously synthesized mosaic HA (mHA) proteins of influenza B viruses which redirect the immune response towards the immuno-subdominant conserved epitopes of the HA *via* sequential immunization. As ~90% of current influenza virus vaccines are manufactured using the inactivated virus platform, we generated and sequentially vaccinated mice with inactivated influenza B viruses displaying either the homologous (same B HA backbones) or the heterologous (different B HA backbones) mosaic HAs. Both approaches induced long-lasting and cross-protective antibody responses showing strong antibody-dependent cellular cytotoxicity (ADCC) activity. We believe the B virus mHA vaccine candidates represent a major step towards a universal influenza B virus vaccine.

## Introduction

Influenza B virus has been a significant public health burden on a global scale (1–3). Approximately 20 to 30% of all clinical influenza cases are caused by influenza B viruses. In some years, they are the most prominent circulating strains of influenza (4–7). Furthermore, influenza B viruses have been described to have significant and disproportionately higher mortality rates compared to influenza A strains in children and infants. For example, during the 2010-2011 epidemic in the United States, influenza B viruses caused 25% of all influenza cases while 38% of all pediatric deaths were attributed to influenza B infection (8). The impact of influenza B virus as a pediatric health problem has also been observed worldwide (7, 9, 10).

Currently, there are two antigenically distinct lineages of influenza B viruses co-circulating in the human population, the B/Yamagata/16/1988-like strains and the B/Victoria/02/1987-like strains, which diverged from a common ancestor in the 1980s (11). The influenza B virus HA contains four major antigenic sites in the globular head domain, the 120 loop, the 150 loop, the 160 loop and the 190 helix (12). These major antigenic sites are immuno-dominant, eliciting most of the HA-specific antibody responses in the host (13, 14). However, the immuno-dominant head domain has high plasticity and is subject to antigenic drift, which allows the virus to escape pre-existing immunity. Therefore, HA-based seasonal influenza virus vaccines need to be re-formulated and re-administered frequently based on surveillance of the circulating strains (15). Although the trivalent influenza virus vaccine (TIV) containing the influenza B virus components of one lineage offers cross-protection in some vaccine recipients (16, 17), a quadrivalent influenza virus vaccine (QIV) containing influenza B components of both lineages started to replace the TIV since 2013 in the United States and also in Europe, aiming for higher vaccine efficacy and broader protection (18–20). However, mismatches between the vaccine strains and circulating strains still substantially decrease vaccine efficacy (21, 22). To overcome such limitations, our group has developed two influenza B virus vaccine strategies, the chimeric HA (cHA) approach and the mosaic HA (mHA) approach (23–28). Both strategies were designed with the rationale of eliciting antibodies against the immuno-subdominant (SD) and more conserved epitopes of the HA protein. The mHA constructs had their immunodominant antigenic sites silenced and they were tested in the recombinant protein vaccine platform.

Because almost 90% of influenza virus vaccines are manufactured using the inactivated vaccine platform, the whole inactivated influenza B viruses (WIVs) displaying the mHAs were used for this study (29). Of note, in addition to the homologous B mHA viruses with the same B/Yamagata/16/1988 HA backbone, we rescued additional heterologous B mHA viruses in different HA backbones covering the two lineages (B/Phuket/3073/2013 and B/Brisbane/60/2008). In mice, we showed that sequential immunization with formaldehyde-inactivated WIVs expressing either the homologous or the heterologous B virus mHAs induced cross-reactive antibody responses against the SD epitopes that were long-lasting and highly cross-protective.

## Materials and Methods

### Cell Culture

Human embryonic kidney 293T (HEK 293T) cells were maintained in Dulbecco’s Modified Eagle Medium (DMEM; Gibco) supplemented with 10% (vol/vol) fetal bovine serum (FBS; Gibco) and 100 units/mL of penicillin, 100 µg/mL of streptomycin (P/S; Gibco). Madin-Darby canine kidney (MDCK) cells were maintained in Minimum Essential Medium (MEM; Gibco) supplemented with 10% (vol/vol) FBS, 100 units/mL of penicillin, 100 µg/mL of streptomycin (P/S; Gibco), 2 mM of L-glutamine (Gibco), 0.15% (w/vol) of sodium bicarbonate (Corning) and 20 mM of 4-(2-hydroxyethyl)-1-piperazineethanesulfonic acid (HEPES; Gibco). Cell lines were maintained at 37°C with 5% CO_2_.

### Recombinant HA Genes and Cloning

The mHA gene segments were based on the HA gene of the B/Yamagata/16/1988, B/Brisbane/60/2008 or B/Phuket/3073/2013 strains. The mosaic HA gene segments were designed by replacing the major antigenic sites of B HA with the corresponding sequences of H5, H8 or H13 viruses (H5, A/Vietnam/1203/2004 H5N1-PR8-IBCDC-RG/GLP; H8, A/mallard/Sweden/24/2002 H8N4; H13, A/black-headed gull/Sweden/1/1999 H13N6) as described before (28). The modified gene segments were obtained as synthetic double-stranded DNA fragments from Integrated DNA Technologies, using the gBlocks^®^ Gene Fragments service, with 15 bp cloning sites specific for the pDZ vector at the 5’ and 3’ ends. The HA segments were subsequently cloned into the pDZ ambisense vector (30) through In-Fusion HD cloning (Takara Bio USA) according to the manufacturer’s protocol. The pDZ B virus mHA plasmids were purified using NucleoBond Xtra Maxi Plus kit (Takara Bio USA) for rescue. Sequences of HA segments were confirmed by Sanger sequencing (Psomagen).

### Rescue of Recombinant Influenza B Viruses

HEK 293T cells were seeded onto poly-D lysine-coated 6-well plates at a ratio of 1:4. The next day, each well of cells was transfected with 2.8 μg of pRS-B/Mal04 7-segment plasmid and 0.7 μg of modified pDZ plasmid encoding the HA segment as well as 0.5 μg of pCAGGS B/Mal04 HA helper plasmid using 20 μL of TransIT LT1 transfection reagent (Mirus Bio). The pRS-B/Mal04 7-segment plasmid drives ambisense expression of the seven gene segments (except HA) of the B/Malaysia/2506/2004 (B/Mal04) mouse-adapted (MA) virus strain and has been described previously (31). Transfected cells were incubated at 37°C for 16 to 20 hours and were then transferred to a 33°C incubator to achieve optimal rescue efficiency. Forty-eight hours post transfection, cells were scraped from the plates and collected along with cell supernatants and briefly homogenized through several syringe strokes. Subsequently, 200 µL of the cell and supernatant mixture were injected into the allantoic cavity of 8-day-old specific pathogen-free (SPF) embryonated chicken eggs (Charles River Laboratories). The eggs were incubated at 33°C. After a 3-day incubation, eggs were cooled to 4°C overnight, and allantoic fluid was harvested and clarified by low-speed centrifugation. A hemagglutination (HA) assay was used to examine the presence of rescued virus using 0.5% turkey red blood cells. HA-positive allantoic fluid samples were used to plaque-purify homogeneous virus clones on confluent MDCK cells. The plaque-purified viruses were further amplified in 10-day old embryonated chicken eggs. RNA was extracted from allantoic fluid containing the plaque-purified viruses using QIAamp Viral RNA Mini Kit (Qiagen). One-step RT-PCR was performed to amplify the HA segment DNA using SuperScript™ III One-Step RT-PCR System with Platinum™ Taq DNA Polymerase (Invitrogen) and HA-specific primers. DNA was purified using NucleoSpin Gel and PCR Clean-up kit (Takara Bio USA) and then sequenced by Sanger sequencing (Genewiz).

### Hemagglutination Inhibition (HI) Assay

Mouse sera were treated with receptor destroying enzyme (RDE; Denka Seiken) to eliminate non-specific inhibitors in the sera. Briefly, serum was mixed with RDE in a 1:3 ratio (vol/vol). RDE-treated samples were incubated at 37°C for 18-20 hours and the reaction was stopped by the addition of 2.5% sodium citrate solution in a 1:3 ratio (vol/vol). The samples were then heat-treated at 56°C for 30 minutes (32). Serum was finally diluted with sterile PBS to reach a final dilution of 1:10. To perform HI assays, virus stocks were diluted in PBS to a final HA titer of 8 HA units (4 wells of HA) per 50 μL sample. Two-fold dilutions (25 μL) of RDE-treated serum in PBS prepared in 96-well V-bottom microtiter plates (Thermo Fisher Scientific) were then combined with 25 μL of the diluted virus. The plates were incubated for 30 minutes at room temperature (RT) to allow HA-specific antibodies to bind to virus (32). Then 50 μL of a 0.5% suspension of turkey red blood cells (Lampire) were added to each well. HI titers were defined as the reciprocal of the highest dilution of serum that inhibited hemagglutination of red blood cells. Mouse antisera against the influenza B viruses used in the HI assays were generated from 6- to 8-week old female BALB/c mice with intranasal infection of either B/Phuket/3073/2013 virus (10^6^ plaque forming units (PFU) per mouse) or reassortant B/Brisbane/60/2008 virus (10^4^ PFU per mouse).

### Preparation of Inactivated Viruses for Vaccination

Plaque-purified and sequence-confirmed B mHA viruses were expanded in 10-day-old SPF embryonated chicken eggs. Pooled allantoic fluids from eggs were treated with 0.03% (vol/vol) formaldehyde at 4°C under continuous shaking. After 72 hours, 25 mL of clarified allantoic fluid were added on top of 5 mL of a 30% sucrose (vol/vol) solution in 0.1 M sodium chloride (NaCl), 1 mM ethylenediaminetetraacetic acid (EDTA) and 10 mM Tris-HCl (pH 7.4) in round-bottom polypropylene copolymer (PPCO) centrifuge tubes (Thermo Fisher Scientific). After ultracentrifugation at 25,000 rpm in a Beckman L7-65 ultracentrifuge (Beckman Coulter) equipped with an SW28 rotor for 2 hours at 4°C, the supernatants were discarded and pellets were recovered in 1 mL of PBS. The total protein concentration of each stock was determined with the Pierce BCA Protein Assay Kit (Thermo Fisher Scientific) according to the manufacturer’s protocol.

### Immunization Studies

For animal immunizations, 6- to 8-week-old female BALB/c mice (Jackson Laboratories) were used for all experiments. Experiments were performed in accordance with protocols approved by the Icahn School of Medicine at Mount Sinai Institutional Animal Care and Use Committee (IACUC). Formaldehyde-inactivated viruses were administered intramuscularly at a dose of 1 μg HA per mouse diluted in a total volume of 100 μL sterile PBS with or without 50 μL of AddaVax adjuvant (Invivogen). PBS-vaccinated mice were included as negative controls. Vaccinations were given in 3- or 4-week intervals. Four weeks after the final immunization, mice were euthanized and blood was obtained by cardiac puncture. Sera were isolated by low-speed centrifugation and were stored at -80°C until use. Mice for longitudinal analysis of antibody dynamics were bled from the submandibular vein on study days 0, 28, 56, 84, 105, 147, 189, 231 and 292.

### Passive Transfer and Challenge Studies

The challenge viruses B/New York/PV01181/2018 and B/New York/PV00094/2017 were isolated by the Personalized Virology Initiative at Icahn School of Medicine at Mount Sinai (ISMMS) and mouse-adapted. Six- to eight-week-old female BALB/c mice (Jackson Laboratories) received 100 or 150 μL of pooled sera *via* intraperitoneal (IP) injection. After 2 hours, mice were infected intranasally with 5 murine 50% lethal doses (5 mLD_50_) of the mouse-adapted B/New York/PV01181/2018 virus, mouse-adapted B/New York/PV00094/2017 virus or B/Lee/1940 virus in a volume of 30 μL of sterile PBS after anesthesia with a ketamine/xylazine cocktail administered intraperitoneally. Animals were monitored for survival and weight loss for 14 days post-challenge and were scored dead and humanely euthanized if they lost more than 25% of their initial body weight.

### Enzyme-Linked Immunosorbent Assay (ELISA)

Recombinant HA proteins were produced as described previously (33). Proteins were coated onto Immulon^®^ 4 HBX 96-well microtiter plates (Thermo Fisher Scientific) at 2 μg/mL in 1x coating buffer (SeraCare Life Sciences Inc.) at 50 μL/well overnight at 4°C. All plates were washed 3 times with 225 μL PBS containing 0.1% (vol/vol) Tween-20 (PBST) and 220 μL blocking solution (3% goat serum, 0.5% non-fat dried milk powder, 96.5% PBST) was added to each well and incubated for 1 hour at RT. Individual serum samples or pooled sera were serially diluted 3-fold in blocking solution followed by a 2-hour incubation at RT. ELISA plates were washed 3 times with PBST and 50 μL of anti-mouse IgG-horseradish peroxidase (HRP) conjugated antibody (Cytiva) was added at a dilution of 1:3,000 in blocking solution. After 1 hour, plates were washed 3 times with PBST and developed using SigmaFast OPD (Sigma-Aldrich) for 10 minutes. Reactions were stopped by adding 50 μL 3M hydrochloric acid (HCl) and absorbance at 492 nm was determined on a Synergy 4 plate reader (BioTek) or similar. For each ELISA plate, the average plus 3 standard deviations of absorbance values of blank wells was used as a cutoff to determine endpoint titers using GraphPad Prism 7.0.

### Antibody Dependent Cell-Mediated Cytotoxicity (ADCC) Reporter Assay

White flat-bottom 96-well plates (Corning) were seeded with 2 × 10^4^ MDCK cells per well. After 24 hours at 37°C, the MDCK cells were washed once with PBS and then infected with influenza B viruses at a multiplicity of infection (MOI) of 5 for a single cycle of virus replication overnight. The next day, the culture medium was removed and 25 μL of assay buffer (RPMI 1640 supplemented with 4% low-IgG FBS [Gibco]) was added to each well. Pooled mouse sera were diluted 1:2 (from a starting dilution of 1:30) in RPMI 1640 medium (Gibco) and added (25 μL per well) to the virus-infected MDCK cells in triplicates. The sera were then incubated with MDCK cells for 30 minutes at 37°C. Genetically modified Jurkat cells expressing the mouse FcγRIV with a luciferase reporter gene under transcriptional control of the nuclear factor-activated T (NFAT) cell promoter were then added to the plate at 7.5 × 10^4^ cells in 25 μL/well (Promega) and incubated for 6 hours at 37°C. At the end of the incubation, a volume of 75 μL of Bio-Glo Luciferase assay reagent (Promega) was added to each well and luminescence was quantified using a Synergy 4 microplate reader (BioTek) using Gen5 2.09 software or similar. Fold induction was calculated as follows: (RLU_induced_−RLU_background_)/(RLU_uninduced_−RLU_background_) and graphed using Prism 7.0.

### Flow Cytometry Analysis

At 56 and 84 days post vaccination, the inguinal lymph nodes were harvested from euthanized mice. Lymph nodes were dispersed into single-cell suspensions by mechanical disruption through 40 μm cell strainer (Fisher Scientific) using syringe plungers into cold PBS. Red blood cells were lysed using ammonium-chloride-potassium (ACK) lysis buffer (0.15 M NH_4_Cl, 10 mM KHCO_3_ and 0.11 mM Na_2_EDTA, pH 7.2-7.4). Cell debris was removed by passing samples through 40 μm cell strainers. Cells were pelleted by centrifugation (450 g, 5 minutes) and re-suspended in FACS buffer (PBS containing 0.1% bovine serum albumin [BSA] and 2 mM EDTA) containing Fc block anti-mouse CD16/CD32 (1:100, eBioscience) for 10 minutes at 4°C and then stained with primary surface antibodies (30 minutes, 4°C). The following antibodies were used for surface staining: Live/Dead™ Near-IR fluorescent reactive dye (Thermo Fisher Scientific), Brilliant Violet 605™ anti-mouse IgD (BioLegend), Pacific Blue™ anti-mouse CD3 (BioLegend), Alexa Fluor^®^ 700 anti-mouse/human CD45R/B220 (BioLegend), PerCP/Cyanine 5.5 anti-MU/HU GL7 antigen (BioLegend) and PE/Cyanine 7 anti-mouse CD38 (BioLegend). Cells were washed in FACS buffer (500 g, 5 minutes) and then incubated in 2% methanol-free paraformaldehyde (PFA) fixation buffer for 20 minutes at 4°C. UltraComp eBeads™ Compensation Beads (Invitrogen) were used for compensation. Samples were washed and re-suspended in FACS buffer and transferred to cluster tubes (Corning) for acquisition. Data was acquired on an BD LSRFortessa cytometer (BD Biosciences) using FACSDiva v7.03 (BD Biosciences) with the appropriate fluorescent minus one (FMO) controls. The data were analyzed using FCS Express 7.0 software.

### Statistics

Statistical analyses were performed using Prism 7.0 (GraphPad). Statistical differences in ELISA data were determined using Kruskal-Wallis one-way analysis of variance (ANOVA) corrected using Dunn’s test for multiple comparisons and unpaired one-tailed t test when comparing two groups. The statistical analyses of FACS data were performed using one-way ANOVA corrected for multiple comparison using the Tukey test. Survival curves were compared using log-rank Mantel-Cox tests against the mock groups (PBS-treated or untreated). Levels of significance are indicated as follows: *P ≤ 0.05; **P ≤ 0.01; ***P ≤ 0.001; ****P ≤ 0.0001; ns, not significant.

## Results

### Inactivated Homologous B mHA Viruses Induced Protective Antibody Responses to the Immuno-Subdominant (SD) Epitopes of the HA

**Figure 1:**
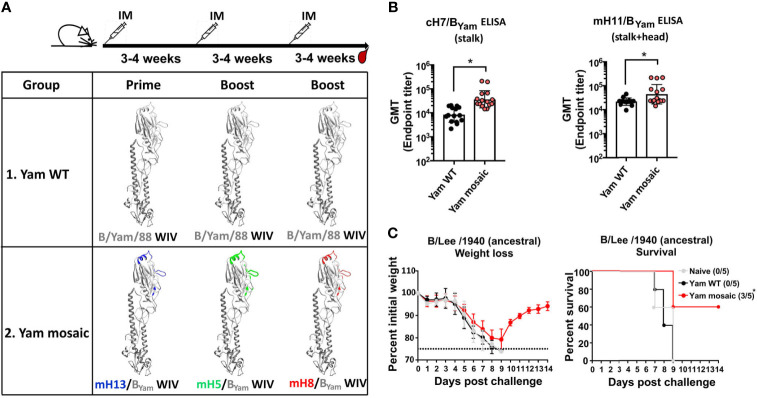
The homologous B mHA viruses induced protective antibody responses to the SD epitopes of the HA. **(A)** Schematic representation of the vaccination groups and regimen [modeled based on the structure of the B/Brisbane/60/2008 HA, PDB accession no. 4FQM (36)]. Residues that were mutated in homologous monomeric B mHAs are shown in different colors which represent corresponding sequences from different influenza A HAs. BALB/c mice (n=15) in the Yam WT group received B/Yam/88 WT HA WIV three times. BALB/c mice (n=15) in the Yam mosaic group received the three B virus mHA WIVs in the order of mH13/B_Yam_ WIV - mH5/B_Yam_ WIV - mH8/B_Yam_ WIV. Each mouse received WIV containing 1 μg of HA. AddaVax was used as the adjuvant and the WIVs were given through intramuscular (IM) administration. Mice were bled 3-4 weeks after the second boost. **(B)** Binding of serum antibodies towards the SD epitopes. A cH7/B_Yam_ protein with a group 2 avian H7 head and the B/Yamagata/16/1988 HA stalk was used to measure stalk-specific antibodies. A mH11/B_Yam_ protein displaying the H11 sequences at the major antigenic sites within the B/Yamagata/16/1988 HA was used to measure antibody binding to SD conserved epitopes in the head and stalk domains. The geometric mean endpoint titer was calculated as the readout. The statistics were calculated using unpaired one-tailed t test (*P ≤ 0.05). **(C)** Cross-protection of serum antibodies against a distant influenza B strain in a passive transfer/viral challenge study. Six- to eight-week-old naïve female BALB/c mice (n=5) received 150 µL of pooled Yam WT, Yam mosaic or naïve sera intraperitoneally. Two hours after the transfer, mice were challenged with 5 mLD_50_ of the B/Lee/1940 strain intranasally. Weight loss and survival of mice were monitored for 2 weeks, with a humane endpoint of >25% loss of the initial weight. In the survival plots, the proportion of surviving animals in each group is shown in parentheses and statistical significance was determined by log-rank Mantel-Cox tests against the naïve group with *P ≤ 0.05.

The mHA approach aims to elicit antibodies not only against the conserved epitopes in the stalk domain but also against those conserved in the head domain outside of the variable antigenic sites. We have shown previously that the vaccine approach with B virus recombinant mHA proteins could induce strong cross-reactive antibody responses and broad protection against viral challenge in mice. Here we assessed whether the same principle holds true for the WIV platform. Three different B mHA viruses in which the major antigenic sites of the HA were replaced by corresponding sequences from H5, H8 or H13 have been constructed and characterized previously (28). The homologous B virus mHAs were based on the B/Yamagata/16/1988 (Yam) HA and designated as mosaic H5/B_Yam_ (mH5/B_Yam_), mosaic H8/B_Yam_ (mH8/B_Yam_), and mosaic H13/B_Yam_ (mH13/B_Yam_) viruses. A virus with unchanged wild-type (WT) B/Yamagata/16/1988 HA served as the control. The B mHA viruses and the virus expressing the WT HA were propagated in embryonated chicken eggs, inactivated with formaldehyde and purified by ultracentrifugation through a sucrose cushion. To normalize HA content in the WIV preparations, we performed an ELISA using a monoclonal antibody (4G12) that broadly binds to an epitope within the stalk region of the influenza B virus HAs (34). The area under the curve (AUC) binding signal was used to calculate HA content relative to the recombinant Yam WT HA protein standard (data not shown). Two groups of 15 mice were vaccinated. Mice in the Yam mosaic group were primed with mH13/B_Yam_ WIV, boosted with mH5/B_Yam_ WIV and boosted again with mH8/B_Yam_ WIV. Mice in the Yam WT group received three doses of the WT WIV. The vaccines were administered with AddaVax, a squalene-based oil-in-water nano-emulsion with a formulation similar to that of MF59, which has been licensed in Europe for adjuvanted influenza virus vaccines (35). The vaccination was performed with a 3- to 4-week interval between doses. Then, mice were bled for serological analysis and passive transfer/viral challenge studies 3 to 4 weeks after the last boost (**Figure 1A**). First, we determined the antibody responses toward the stalk domain of HA by ELISAs using the chimeric H7/B_Yam_ (cH7/B_Yam_) recombinant protein. The cH7/B_Yam_ has an H7 head domain on top of the stalk domain of the Yam HA. We observed that the Yam mosaic group developed significantly higher levels of stalk-reactive IgG than the Yam WT group (**Figure 1B**). Additionally, the antibody levels against the SD epitopes in both stalk and head domains were assessed by ELISAs using the mosaic H11/B_Yam_ (mH11/B_Yam_) protein displaying the H11 sequences at the major antigenic sites of the Yam HA. As expected, in comparison to the Yam WT group, the Yam mosaic group reached higher IgG titer against the mH11/B_Yam_ than the Yam WT group (**Figure 1B**). Next, we determined the ability of the antibodies induced by the Yam mHA vaccines to confer cross-protection against lethal challenge of the antigenically divergent ancestral B/Lee/1940 virus strain. Groups of 5 naïve mice received 150 μL per mouse of pooled sera intraperitoneally and were challenged 2 hours later with 5 mLD_50_ of B/Lee/1940 virus. Mice were observed daily for weight loss and mortality for 14 days post-challenge. All mice challenged with B/Lee/1940 virus showed substantial weight loss (**Figure 1C**). Of note, all mice in the naïve group and the Yam WT group succumbed to infection by day 9 post-challenge, whereas 60% of the animals in the Yam mosaic group survived. This showed that serum antibodies elicited by the homologous B virus mHA WIVs induced better cross-protection against challenge with an antigenically distinct virus than that provided by immunization with the WT WIV.

### Rescue and Characterization of Heterologous B mHA Viruses

**Figure 2:**
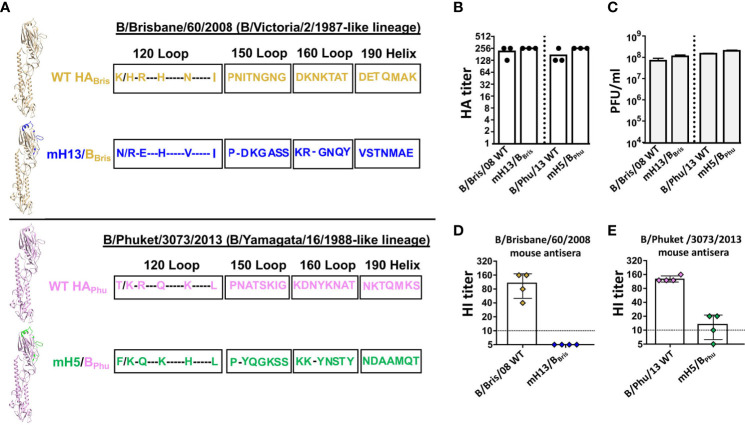
Rescue and characterization of the heterologous B mHA viruses. **(A)** Schematic representation of the heterologous B mHAs design [modeled based on the structure of the B/Brisbane/60/2008 HA, PDB accession no. 4FQM (36)]. The left upper panel shows monomeric B/Brisbane/60/2008 WT HA and mHA. Residues that were mutated in monomeric mH13/B_Bris_ are shown in blue. The left lower panel shows monomeric B/Phuket/3073/2013 WT HA and mHA. Residues that were mutated in monomeric mH5/B_Phu_ are shown in green. Amino acid sequences of the major antigenic sites of the B virus mHA are aligned with the corresponding sequences of the WT HA. The sequence alignment was performed with the HAs of B/Brisbane/60/2008, mH13/B_Bris_, B/Phuket/3073/2013 and mH5/B_Phu_ (H5: A/Vietnam/1203/04 H5N1-PR8-IBCDC-RG/GLP; H13: A/black-headed gull/Sweden/1/1999 H13N6). **(B)** HA titers and **(C)** plaque-forming units (PFU) of influenza B viruses grown from eggs. Allantoic fluid virus stocks were titrated by HA assay and plaque assay on MDCK cells in triplicate. Bars represent the mean ± SD. **(D)** HI assay using the indicated viruses and antisera of mice (n=4) raised against the reassortant B/Brisbane/60/2008. Symbols representing individual mice are color coded and the bars show the mean ± SD value of each group. **(E)** HI assay using the indicated viruses and antisera of mice (n=4) raised against the B/Phuket/3073/2013. Symbols representing individual mice are color coded and the bars show the mean ± SD value of each group.

To improve the B virus mHA vaccine approach by introducing different HA backbones for the benefit of enriching cross-lineage antibodies, we rescued B mHA viruses either in the B/Phuket/3073/2013 (Phu, B/Yamagata/16/1988-like lineage) or the B/Brisbane/60/2008 (Bris, B/Victoria/2/1987-like lineage) HA backbones. We replaced the major antigenic sites with the corresponding sequences from H5 (Phu HA) or H13 (Bris HA) (**Figure 2A**). Those heterologous B mHA viruses were designated as mH5/B_Phu_ and mH13/B_Bris_ respectively. The amino acid sequence alignment of the B/Phuket/3073/2013 or B/Brisbane/60/2008 HA with mH5/B_Phu_ or mH13/B_Bris_ is shown in **Figure 2A**. Two viruses with WT B/Phuket/3073/2013 (Phu) or WT B/Brisbane/60/2008 (Bris) HA were also rescued as controls. The newly generated heterologous WT HA and B mHA viruses grew well in eggs, reaching a hemagglutination titer above 1:128 (**Figure 2B**) and an infectious titer of approximately 10^8^ PFU/mL (**Figure 2C**). The major antigenic sites elicit most of the hemagglutination inhibition (HI) antibodies as they are relatively close to the receptor binding site (RBS) (13). To confirm the ablation of the original antigenic sites in the B virus mHAs, HI assays were performed using mouse sera raised against viruses with the WT HAs. As expected, mouse sera generated using the WT B/Brisbane/60/2008 HA virus inhibited hemagglutination strongly against the same virus. In contrast, no detectable HI reactivity was measured against the mH13/B_Bris_ (**Figure 2D**). Similarly, the mouse sera raised against B/Phuket/3073/2013 virus showed high HI titers against the same virus, while the HI titers against the mH5/B_Phu_ virus were nearly 10-fold lower (**Figure 2E**). These results confirmed that the original major antigenic sites had been successfully silenced in the two heterologous B mHA viruses.

### Inactivated Heterologous B mHA Viruses Induced Protective Antibody Responses to the SD Epitopes of the HA

**Figure 3:**
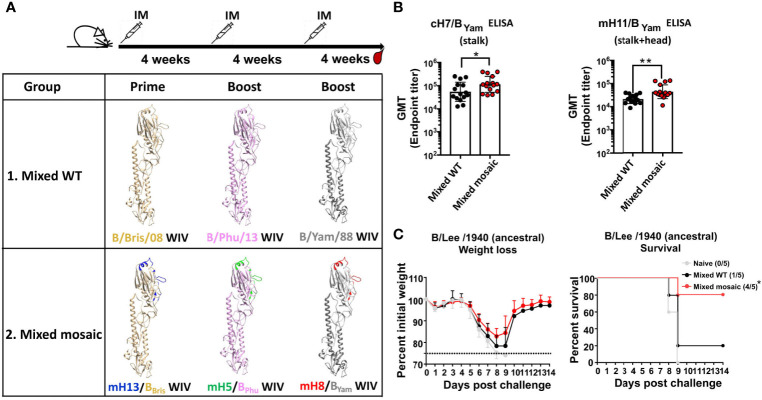
The heterologous B virus mHA viruses induced protective antibodies responses to the subdominant epitopes of the HA. **(A)** Schematic of the vaccination groups and regimen [modeled based on the structure of the B/Brisbane/60/2008 HA, PDB accession no. 4FQM (36)]. Residues that were mutated in the heterologous monomeric B mHAs are indicated in different colors, representing corresponding sequences from different influenza A HAs. BALB/c mice (n=15) in the Mixed WT group received B/Bris/08 WT HA WIV followed by B/Phu/13 WT HA WIV and then B/Yam/88 WT HA WIV. Mice (n=15) in the Mixed mosaic group received the B virus mHA WIV sequentially as follows: mH13/B_Bris_ WIV - mH5/B_Phu_ WIV - mH8/B_Yam_ WIV. Each mouse received WIV containing 1 μg of HA. Each vaccination was given at 4-week intervals. AddaVax was used as the adjuvant and the WIVs were administered through intramuscular (IM) injection. Mice were bled 4 weeks after the second boost. **(B)** Binding of serum antibodies towards the SD epitopes. The cH7/B_Yam_ protein and the mH11/B Yam protein were used in ELISAs as described in **Figure 1**. The geometric mean endpoint titer (GMT) was calculated as the readout. The statistics were performed using the unpaired one-tailed t test (*P ≤ 0.05 and **P ≤ 0.01). **(C)** Cross-protection of serum antibodies against a distant influenza B strain in a passive transfer/viral challenge study. Naïve BALB/c mice (n=5) received 100 μL of pooled Mixed WT, Mixed mosaic or naïve sera intraperitoneally. Two hours after transfer, mice were challenged with 5 mLD_50_ of the B/Lee/1940 strain intranasally. Weight loss and survival of mice were monitored for 2 weeks, with a humane endpoint of >25% loss of the initial weight. In the survival plots, the proportion of surviving animals in each group is shown in parentheses and statistical significance was inferred by log-rank Mantel-Cox tests against the naïve group with *P ≤ 0.05.

Humans are exposed to various strains of influenza B viruses *via* vaccinations and infections throughout life. Consequently, people of older age tend to have more stalk-specific antibodies than younger people (37). One way to demonstrate the value of the mosaic HA approach is to show that the heterologous B virus mHAs would elicit more antibodies to the SD epitopes than the heterologous WT HA. Therefore, we performed a similar immunization study as described in **Figure 1** using formaldehyde-inactivated WIVs (normalized to 1 μg HA per mouse) (**Figure 3A**). The WT group was primed with Bris WIV and subsequently boosted with the Phu WIV followed by the Yam WIV (“Mixed WT”). The B virus mHA group was primed with mH13/B_Bris_ WIV and boosted with mH5/B_Phu_ WIV then mH8/B_Yam_ WIV (“Mixed mosaic”). Three immunizations were given with a 4-week interval between doses. Each mouse was vaccinated with AddaVax as the adjuvant. Again, 4 weeks after the last immunization, mice were terminally bled to isolate sera. We performed ELISAs using cH7/B_Yam_ (stalk) or mH11/B_Yam_ (stalk and head) proteins to measure antibodies against the SD epitopes. In comparison to the Mixed WT group, the Mixed mosaic group demonstrated higher levels of antibodies targeting the SD epitopes of the B HA (**Figure 3B**). Then, we determined antibody-mediated cross-protection in mice by passive transfer and viral challenge study using the B/Lee/1940 strain. Groups of 5 naïve mice received 100 μL per mouse of pooled sera intraperitoneally and were lethally challenged 2 hours later with a dose of 5 mLD_50_. All mice that received naïve mouse sera succumbed to infection by day 9. Importantly, 80% of animals in the Mixed mosaic group survived, while only 20% in the Mixed WT group survived (**Figure 3C**). These data indicated that the heterologous B virus mHA WIVs conferred better cross-protection than the heterologous WT HA WIVs, emphasizing that the B virus mHAs can induce cross-protective antibodies more efficiently than the heterologous WT HAs.

### Head-to-Head Comparison of the Homologous B Virus mHA With the Heterologous B Virus mHA Vaccine Candidates

**Figure 4:**
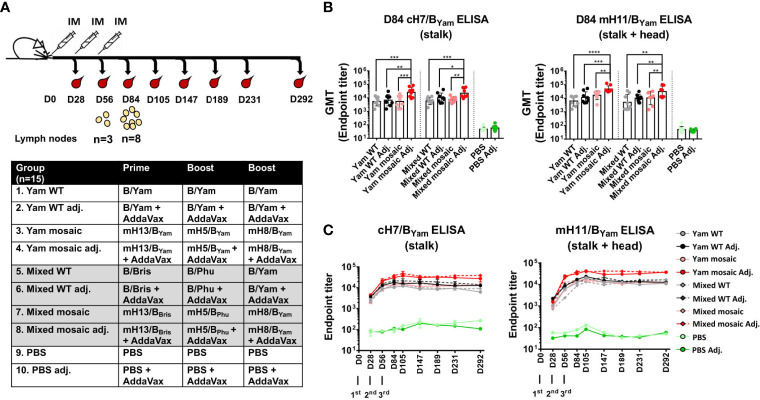
Immunogenicity of homologous versus heterologous B virus mHA constructs. **(A)** Vaccination groups and regimen. The groups (n=15) include homologous WT B/Yam (Yam WT), homologous B virus mHA (Yam mosaic), heterologous WT HA (Mixed WT), heterologous B virus mHA (Mixed mosaic) and PBS, each in the presence or absence of the adjuvant (AddaVax; Adj.). Mice were immunized with inactivated WIV at 4-week intervals *via* intramuscular injection. Two subsets of animals were euthanized on D56 and D84 to analyze germinal center reactions in the draining lymph nodes. Another subset of animals was terminally bled for serology. The remaining animals were kept until D292 and bled periodically to examine the longevity of antibody responses. **(B)** Serum IgG directed to the SD epitopes on D84. ELISAs were performed as described previously. The statistical analysis was performed using one-way ANOVA corrected for multiple comparison using Dunnett’s test (*P ≤ 0.05; **P ≤ 0.01; ***P ≤ 0.001; ****P ≤ 0.0001). **(C)** Longevity of antibody responses. Pooled mouse sera from each group obtained on the indicated days were tested with a technical duplicate. Endpoint titers were plotted.

We have shown that both homologous and heterologous B virus mHA WIVs are efficient at inducing SD epitope-specific antibodies. To determine if one approach is superior than the other in our mouse model, we performed a head-to-head comparison of sequential immunizations using homologous B mHA viruses (mH13/B_Yam_ - mH5/B_Yam_ - mH8/B_Yam_, Yam mosaic) versus heterologous B mHA viruses (mH13/B_Bris_ -mH5/B_Phu_ - mH8/B_Yam_, Mixed mosaic). The homologous WT HA virus (Yam WT) group, heterologous WT HA viruses (B/Bris - B/Phu - B/Yam, Mixed WT) group and PBS-treated group were included as controls (**Figure 4A**). The immunization was performed in the presence or absence of an adjuvant (AddaVax). Mice were bled periodically for serological assays. In fact, the animals were followed up to 292 days and bled on days 28, 56, 84, 105, 147, 189, 231 and 292 to assess the longevity of antibodies against the SD epitopes. Again, we determined the antibody responses of day 84 (D84) sera toward the SD epitopes by ELISA using cH7/B_Yam_ and mH11/B_Yam_ proteins (**Figure 4B**). Both mHAs vaccine strategies induced robust levels of SD epitope-reactive IgGs when the vaccines were adjuvanted. Additionally, we did not see differences in antibody titers between Yam mosaic and Mixed mosaic groups. Furthermore, as a universal vaccine candidate should provide durable protection, we performed a longitudinal analysis of antibody responses against the SD epitopes (**Figure 4C**). We found that: (i) the adjuvanted Yam mosaic and Mixed mosaic vaccines elicited the highest antibody titers among all the vaccines, (ii) antibody titers against SD epitopes of HA increased substantially after booster immunization and reached a plateau by day 105 and (iii) antibody titers against the SD epitopes remained at the plateau level up to day 292.

**Figure 5:**
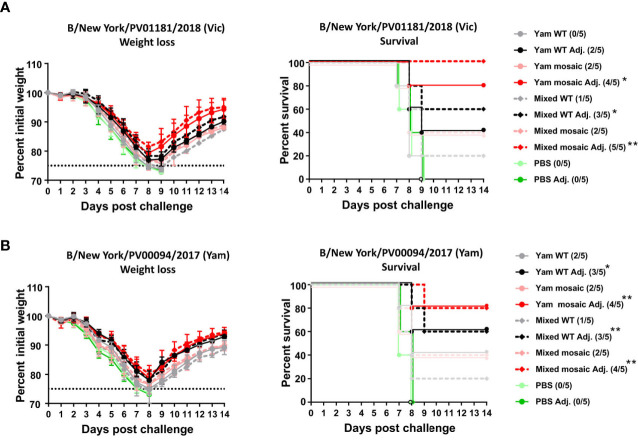
Protection of D292 serum antibodies induced by homologous versus heterologous B virus mHAs vaccination. **(A)** Weight loss and survival of vaccination groups challenged with B/New York/PV01181/2018 virus. Mice (n = 5) received 100 μL of D292 pooled sera intraperitoneally and were challenged intranasally with 5 mLD_50_ of challenge virus. Weight loss and survival of mice were monitored for 2 weeks with a humane endpoint of >25% loss of the initial weight. In the survival plots, the proportion of surviving animals in each group is shown in parentheses and statistical significance was inferred by log-rank Mantel-Cox tests against the corresponding control group (PBS or PBS Adj.) with *P ≤ 0.05 and **P ≤ 0.01.**(B)** Weight loss and survival of vaccination groups challenged with B/New York/PV00094/2017 virus. Mice (n = 5) received 100 μL of D292 pooled sera intraperitoneally and were challenged intranasally with 5 mLD_50_ of challenge virus. Weight loss and survival of mice were monitored for 2 weeks with a humane endpoint of >25% loss of the initial weight. In the survival plots, the proportion of surviving animals in each group is shown in parentheses and statistical significance was inferred by log-rank Mantel-Cox tests against the corresponding control group (PBS or PBS Adj.) with *P ≤ 0.05 and **P ≤ 0.01.

Next, we performed passive transfer/viral challenge studies using the day 292 (D292) sera to evaluate long-term protection. The challenge studies were performed using two recently circulating influenza B viruses representing the B/Victoria/2/1987-like lineage (B/New York/PV01181/2018) and the B/Yamagata/16/1988-like lineage (B/New York/PV00094/2017) that had been isolated from clinical samples and mouse-adapted. Sera were pooled within groups and then transferred into naïve mice. Each naïve mouse received 100 μL of pooled sera intraperitoneally. Two hours after serum transfer, mice were infected with a dose of 5 mLD_50_ of either virus. Challenge with the B/Victoria/2/1987-like strain B/New York/PV01181/2018 resulted in substantial weight loss in all groups (**Figure 5A**). All mice in the PBS control group succumbed to infection. Although more than 50% of the mice in all unadjuvanted groups succumbed to infection by day 9 post-challenge, the mosaic groups exhibited slightly better survival rates (Yam WT: 0%; Yam mosaic: 40%; Mixed WT: 20%; Mixed mosaic: 40%). In contrast, 80% of mice in the adjuvanted Yam mosaic group and 100% of mice in the adjuvanted Mixed mosaic group survived. The adjuvanted Yam WT group and Mixed WT group showed relatively lower survival rates (40% and 60%, respectively) than the adjuvanted mosaic groups (Yam mosaic: 80%; Mixed mosaic: 100%). No statistical difference in survival was detected in animals receiving Yam mosaic and Mixed mosaic vaccines. Lethal challenge with the B/Yamagata/16/1988-like virus B/New York/PV00094/2017 produced similar results (**Figure 5B**). All animals that were given PBS group sera succumbed to infection. For the unadjuvanted vaccination groups, more than 50% of mice in each group succumbed to infection by day 9 post-challenge. While 80% of mice that received sera from mice vaccinated with adjuvanted Yam mosaic or Mixed mosaic survived, 60% of mice that received adjuvanted Yam WT or Mixed WT sera survived. Similarly, no statistical difference in survival was detected in animals receiving the sera from Yam mosaic and Mixed mosaic vaccinations. In summary, both homologous and heterologous B virus mHA vaccine candidates elicited comparable antibody titers against the SD epitopes of HA and provided similar protection *in vivo*. Sequential immunization with mHA vaccines provided better cross-protection against divergent viruses than immunization with the WT HA viruses.

At last, we examined germinal center B cells in the draining lymph nodes as we were exploring the mechanism of action of the B virus mHA constructs. We took inguinal lymph nodes from a subset of animals at day 56 (D56, n=3) and day 84 (D84, n=8) and analyzed the relative abundance of germinal center B cells by flow cytometry (38–40). We observed that the sequential immunization with B virus mHA vaccines appeared to stimulate a stronger germinal center reaction, an effect which AddaVax strengthened significantly, even activating germinal center reactions when administered by itself in the PBS group (**Supplementary Figure 1**). We postulate that the sequential immunization of the B virus mHA constructs drives the memory B cells to re-enter a germinal center to undergo somatic hypermutations and affinity maturation as we expect the B cell receptors (BCRs) to the SD epitopes would have lower affinity to their epitopes than those that react to the immuno-dominant epitopes in the WT HA constructs. The MF59-like adjuvant AddaVax may increase antigen uptake or the adjuvant itself may promote a potent T follicular helper cell (Tfh) response and the persistence of germinal center B cells (41). Interestingly, the frequency of germinal center B cells appeared to correlate with the levels of antibodies targeting the SD epitopes.

### Both Homologous and Heterologous mHA Vaccines Induced Non-HI Active Antibodies With Antibody-Dependent Cellular Cytotoxicity (ADCC) Activity

**Figure 6:**
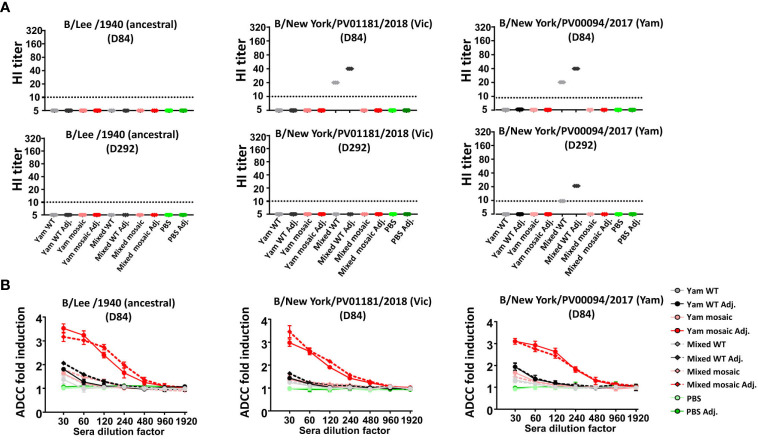
Activity of serum antibodies induced by homologous versus heterologous B virus mHA constructs. **(A)** HI activity of D84 (upper panel) and D292 (lower panel) pooled sera against influenza B viruses including B/Lee/1940, B/New York/PV00094/2017 and B/New York/PV01181/2018. **(B)** ADCC activity of D84 pooled sera in a reporter assay. To perform ADCC reporter assay, MDCK cells were infected with each virus at an MOI of 5 with single-cycle replication. Fold induction of the reporter signal from the sera over those from the blanks were analyzed. Data points represent mean ± SD of pooled sera from 8 mice measured in technical triplicates.

Next, we sought to determine the activity of the antibodies induced by the vaccine candidates. HI activity is a known correlate of protection, with a titer of 1:40 considered to confer 50% protection against seasonal influenza in human adults (42). Consistently with our previous data (28), both B virus mHA vaccination sera showed no detectable HI titers against diverse influenza B viruses including B/Lee/1940, B/New York/PV01181/2018 and B/New York/PV00094/2017 (**Figure 6A**). Not surprisingly, we found the Mixed WT groups elicited detectable levels of HI titers against the B/New York/PV01181/2018 and B/New York/PV00094/2017 viruses. This result was expected because the B/Brisbane/2008 and B/Phuket/3073/2013 strains in the Mixed WT group are phylogenetically close to the B/New York/PV01181/2018 and B/New York/PV00094/2017 virus isolates, respectively. However, we observed that the HI-active antibodies against the B/New York/PV01181/2018 and B/New York/PV00094/2017 viruses on D292 declined significantly compared to those on D84, unlike the long-lasting antibodies targeting the SD epitopes (**Figure 4C**). These data suggested that the protection conferred by the B virus mHA vaccination approach was not through HI. Previously, we have described that Fc-mediated effector functions (such as ADCC) can be one of the mechanisms by which stalk-specific antibodies contribute to protection *in vivo* independently of HI activity (43–46). Therefore, we tested whether serum antibodies induced by B virus mHAs engaged in Fc-mediated effector functions using an ADCC reporter assay. We observed that D84 pooled sera of adjuvanted Yam mosaic or Mixed mosaic vaccinated mice showed strong induction of ADCC activity when incubated with MDCK cells infected with B/Lee/1940, B/New York/PV00094/2017 or B/New York/PV01181/2018 (**Figure 6B**). In contrast, the adjuvanted Yam WT or Mixed WT group elicited limited levels of antibodies with ADCC activity. Therefore, the B virus mHA vaccination approach likely conferred protection through non-HI-active antibodies that trigger Fc-mediated effector functions.

## Discussion

The development of universal influenza virus vaccines that can provide durable protection against multiple strains is one of the most critical global public health priorities. To date, the vast majority of these efforts have focused on influenza A viruses. Given the significant clinical burden and economic impact that influenza B viruses pose on the global population, more efforts should be made to design or refine influenza B virus vaccines. Importantly, in contrast to influenza A viruses, influenza B viruses lack animal reservoirs, except for rare spillovers into marine mammals (47). Another notable difference between influenza A and B viruses is that the evolutionary rate of influenza B viruses is much lower than that of influenza A viruses (48, 49). Both the host restriction and the low evolutionary rate allow for the possibility of eradicating influenza B viruses. Here, a broadly protective inactivated virus vaccine strategy using the B virus mosaic HAs is described, which builds on an earlier mosaic B virus HA protein study (28). The approach is based on the antigenic silencing of the major antigenic sites of the type B HA by replacing them with exotic sequences from HAs of avian influenza A viruses (H5, H8 and H13), yielding mosaic HAs. Compared to the recombinant B mHA proteins, the inactivated B mHA vaccines will provide additional protection mediated by other viral components such as the neuraminidase (NA) and internal proteins. Importantly, the inactivated virus vaccine platform is compatible with most of the influenza virus vaccine production capacity. As hypothesized, sequential vaccination with WIVs expressing the B virus mosaic HAs induced higher cross-reactive antibodies against the conserved epitopes than immunization with WIVs expressing the WT HAs. The mHA approach relies on the replacement of the hypervariable immunodominant epitopes. This can be achieved by either using exotic epitopes from other HA subtypes (as shown in this study) or by introducing irrelevant epitopes such as those consisting of glycines and/or alanines. The latter strategy would very much increase the number of possible mHAs.

Of note, the adjuvant AddaVax, an oil-in-water emulsion adjuvant, had a positive role in inducing antibodies against the conserved epitopes of HA. When adjuvanted with AddaVax, the B virus mHA vaccines induced significantly higher antibody titers than the unadjuvanted B virus mHA vaccines. This effect, to a lesser extent was also observed in WT HA vaccine groups. Interestingly, we also observed that sequential immunization with the B virus mHA vaccines adjuvanted with AddaVax generated higher levels of germinal center B cell reactions than non-adjuvanted B virus mHA vaccines. There seems to be a correlation between the antibody titers against the SD epitopes and the frequency of germinal center B cells. We speculate that this positive effect on germinal center reaction in B virus mHA immunization is due to a subset of recalled memory B cells with relatively low affinity re-entering germinal centers to undergo further somatic hypermutation and affinity maturation. AddaVax seems to facilitate a persistent activation of germinal centers, subsequently helping to boost the antibodies targeting SD epitopes. Furthermore, our results demonstrated that antibody titers against SD epitopes (e.g., stalk-specific antibodies) can be maintained at high levels over a long period of time in mice. We did not observe differences of SD epitope-specific antibodies induced by the homologous and heterologous B virus mHA vaccines. But the protection provided by the heterologous B virus mHAs in the B/Lee/1940 and B/Victoria/2/1987-like B/New York/PV01181/2018 challenge studies seemed to be better by a narrow margin than that provided by the homologous B virus mHAs (**Figures 1**, **3**, **5**). It is also worth noting that in the mouse study, we were comparing the B virus mHA vaccine with their corresponding WT HA constructs. In fact, the current seasonal influenza virus vaccine is a single immunization of a multi-valent vaccine instead of sequential vaccination with heterologous strains. Therefore, it is likely that one or two immunizations with the B virus mHA vaccines in the primed human population would enrich even more SD epitope-specific antibodies than one shot of TIV or QIV. Future clinical studies would be needed to recapitulate these findings and to evaluate the B virus mHA in combination with different adjuvants.

The B virus mHA approach using the inactivated virus vaccine platform induced broadly-reactive antibodies without detectable HI activities. We expect these antibodies are also non-neutralizing based on our previous study (28). Serum antibodies generated by a sequential vaccination with B virus mHA WIVs was highly active in an ADCC reporter assay that measures Fc-mediated effector functions. This was described as an important mechanism of protection for HA stalk-reactive antibodies of influenza A viruses (43, 44). Here we showed that AddaVax enhanced ADCC activity of the antibodies resulting from vaccination with B virus mHAs. Although it was not measured in this study, a robust induction of IgG2a is expected in mice vaccinated with B mHA vaccines, as it was observed from our preclinical study involving the influenza A mHA vaccines (50). In contrast, sequential vaccination with WT HAs elicited limited levels of antibodies with ADCC reporter activity. Instead, the heterologous WT HA WIVs induced HI-active antibodies to the virus substrates due to the presence of similar immuno-dominant antigenic sites. However, the HI-active antibodies were not as long-lasting as the antibodies targeting the SD sites, explaining the lower protection of WT HA WIV sera as compared to mHA WIV sera collected at D292 in passive transfer/viral challenge studies. Consistent with what was observed in mice, a recent phase I clinical trial demonstrated that stalk-specific antibodies induced by sequential vaccination of group 1 cHA vaccines were long-lived (26).

Currently, the inactivated split-virion vaccine is most commonly used due to the relatively low production costs and high safety. Recombinant HA subunit vaccines are also available to effectively focus on HA-mediated protection. However, the production capacity of the subunit vaccine is limited (29). Despite the possible loss of immunogenicity, split-virion vaccines and subunit vaccines are used more frequently than the whole-virion vaccines in humans due to their reduced reactogenicity (51–53). Moreover, the recommended vaccine strains sometimes replicate poorly in eggs. Many passages of the vaccine strain in egg might be necessary to achieve high titers, yielding adaptive mutations. These changes, often within the major antigenic sites, can alter the antigenicity of the HAs, resulting in an antigenic mismatch of the vaccine and the circulating strain (54). Of note, the mHA vaccine approach provides durable and broad protection and is suitable for multiple vaccine platforms including inactivated, live attenuated and recombinant HA vaccines. Here, we only investigated the WIV as a platform. It would be of interest in the future to compare the B virus mHA WIV vaccine versus mHAs vaccine in other platforms. Finally, as we only show the activity of serum polyclonal antibodies in this study, further studies will focus on isolating and characterizing monoclonal antibodies targeting the SD epitopes after sequential immunization using mHA vaccines. The interaction of such monoclonal antibodies against the SD HA head epitopes will be studied by structural biology techniques including X-ray crystallography and cryo-EM.

## Data Availability Statement

The raw data supporting the conclusions of this article will be made available by the authors, without undue reservation.

## Ethics Statement

The animal study was reviewed and approved by Icahn School of Medicine at Mount Sinai Institutional Animal Care and Use Committee (IACUC).

## Author Contributions

WS and PP initiated this study. WS, PP, FK, AG-S, and YL designed the experiments. YL, WS, and IG-D performed the experiments. YL and WS analyzed the data. SS, JT, and VS provided protein and virus reagents. YL, WS, and PP wrote the manuscript. All authors contributed to the article and approved the submitted version.

## Funding

This work was partially funded by NIH (Centers of Excellence for influenza Research and Response, CEIRR,75N93021C00014, PP, FK, AG-S), (NIAID grant P01 AI097092-07, PP, AG-S), (NIAID grant R01 AI145870-03, PP) and by the Collaborative Influenza Vaccine Innovation centers (CIVICs) contract 75N93019C00051 (PP, FK, AG-S).

## Conflict of Interest

The Icahn School of Medicine at Mount Sinai has filed patent applications entitled “INFLUENZA VIRUS HEMAGGLUTININ PROTEINS AND USES THEREOF” which names PP, FK, AG-S as inventors.

The remaining authors declare that the research was conducted in the absence of any commercial or financial relationships that could be construed as a potential conflict of interest.

The handling Editor declared a past co-authorship with one of the authors FK.

## Publisher’s Note

All claims expressed in this article are solely those of the authors and do not necessarily represent those of their affiliated organizations, or those of the publisher, the editors and the reviewers. Any product that may be evaluated in this article, or claim that may be made by its manufacturer, is not guaranteed or endorsed by the publisher.

## Supplementary Material

Supplementary Figure 1 Frequency of germinal center B cells. Inguinal lymph nodes were collected on D56 **(A)** and D84 **(B)** and frequency of germinal center B cells (CD3^-^B220^+^IgD^-^GL7^+^CD38^low^) was measured by FACS. The statistical analysis was performed using one-way ANOVA corrected for multiple comparison using the Tukey test (*P ≤ 0.05; **P ≤ 0.01; ***P ≤ 0.001; ****P ≤ 0.0001; ns, not significant).
