# Supplementary figures and images for: Mosaic Hemagglutinin-Based Whole Inactivated Virus Vaccines Induce Broad Protection Against Influenza B Virus Challenge in Mice
Source: Front Immunol. 2021 Sep 16;12:746447. doi: 10.3389/fimmu.2021.746447 (PMC8481571; doi:10.3389/fimmu.2021.746447)

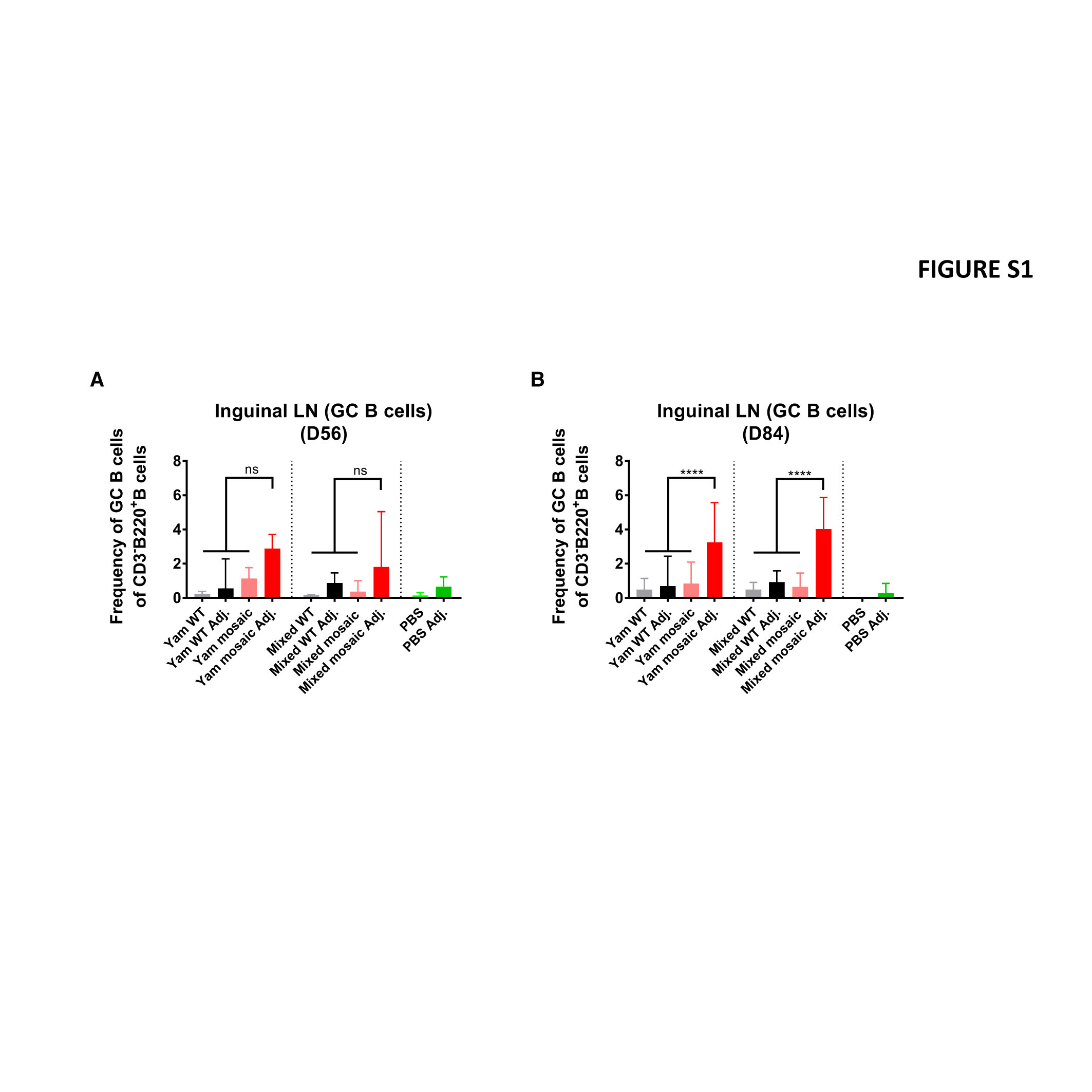

Supplement: Supplementary Figure 1 — Frequency of germinal center B cells. Inguinal lymph nodes were collected on D56 (A) and D84 (B) and frequency of germinal center B cells (CD3-B220+IgD-GL7+CD38low) was measured by FACS. The statistical analysis was performed using one-way ANOVA corrected for multiple comparison using the Tukey test (*P ≤ 0.05; **P ≤ 0.01; ***P ≤ 0.001; ****P ≤ 0.0001; ns, not significant). [file Image_1.jpeg]
